# Cluster munitions: military use and civilian health hazards

**DOI:** 10.2471/BLT.17.202481

**Published:** 2018-06-04

**Authors:** Jawad Fares, Youssef Fares

**Affiliations:** a Department of Neurosurgery, Lebanese University, Beirut, Lebanon.

Official reports from the Syrian Arab Republic and Yemen indicate the use of cluster munitions in civilian areas.^1^ Cluster munitions are a form of air-dropped or ground-launched explosive weapons that eject numerous smaller submunitions. These weapons have military value, because of their wide dispersal, versatility and effectiveness against targets that move or do not have precise locations, such as moving troops or vehicles. Submunitions are designed to explode on impact; however, when they fail to explode as expected, the duds usually remain hazardous and will detonate when touched or disturbed. Globally, submunitions have taken more civilian lives and limbs after the cluster munition strikes than during attacks.^2^ The impact of such submunitions goes beyond civilian casualties, as extensive submunition contamination can have far-reaching socioeconomic and environmental consequences, hindering post-conflict reconstruction and development.

**Fig. 1:**
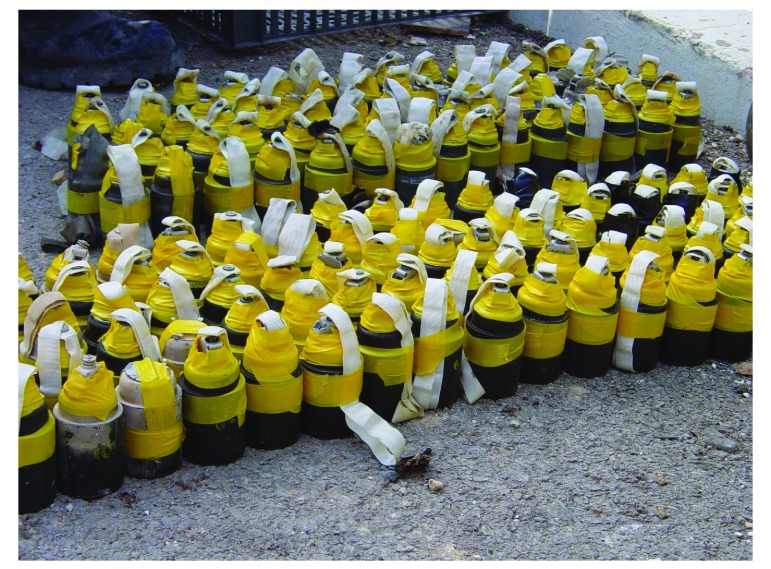
Undetonated submunitions collected from southern Lebanon, 2010

Estimations indicate that during the July–August 2006 hostilities between Lebanon and Israel,^3^ 4.6 million submunitions were released over Lebanon, 1 million of which remained unexploded (Fig. 1).^4^ After the war, these unexploded duds continued to cause injuries and deaths when civilians returned to their lands and homes. The war provided momentum for a campaign to ban cluster munitions and on 1 August 2010 the Convention on Cluster Munitions entered into force. To date 120 countries have committed to the goals of this convention, with 103 full parties and 17 signatories.^5^ This international treaty prohibits all use, production, transfer and stockpiling of cluster munitions. In addition, the convention establishes a cooperation framework to assist victims and their communities, and to provide clearance of contaminated areas, awareness raising on risks and safe destruction of stockpiles.^5^

Here we highlight the effects of cluster munitions and call for action against their use.

In Lebanon, more than 500 casualties have been recorded since hostilities ended in 2006, with craniofacial injuries (7%; 29/356), abdominal and thoracic injuries (28%; 101/356) and upper (62%; 220/356) and lower limb (83%; 294/356) amputations due to submunition detonation.^6^ These injuries cause significant disability, and treatment is complicated by wound infections caused by organisms including *Pseudomonas* (5%; 18/350), *Escherichia coli* (5%; 16/350) and *Candida* (3%; 9/350), sometimes leading to necrosis (5%; 18/350).^7^ Many injured people were also diagnosed with post-concussion syndrome (30%; 34/112) and traumatic brain injuries, both penetrating (24%; 7/29) and closed (38%; 11/29).^8^^,^^9^ Survivors often develop long-term psychosocial and mental health sequelae, predominantly post-traumatic stress disorder (43%; 105/244) and depression (63%; 154/244).^10^^,^^11^

Cluster munitions also contributed to internal displacement. Contaminated areas include agricultural fields and cultural and historic monuments.^12^ Fear of cluster munitions forced populations to move into subsidiary locations to avoid the unexploded ordnances.^12^

In 2015, the International Committee of the Red Cross signed an agreement with the Faculty of Medicine of the Lebanese University to give a module on the clinical management of the war-wounded, but more can be done. The World Health Organization and other health actors such as Médecins Sans Frontières and the International Committee of the Red Cross, could send health professionals and trauma specialists, who are familiar with war injuries, to build the capacity of local health workers in affected countries such as Lebanon, Syrian Arab Republic and Yemen. These organizations could also cooperate with the health ministries of affected countries to develop awareness programmes on submunitions and other unexploded ordnances of war.

Most of the cluster munitions used in the Syrian Arab Republic (since 2012) and in Yemen (since March 2015), are manufactured in Brazil, Russian Federation, United Kingdom of Great Britain and Northern Ireland and United States of America.^13^ Until early 2017, Human Rights Watch had documented that cluster munitions were used in 18 attacks in Yemen, leading to 21 civilian casualties and 74 injuries.^14^ In the Syrian Arab Republic, the number of attacks and casualties has not yet been determined.

The United Nations Mine Action Service, Security Council, General Assembly and Human Rights Council, need to take action to control the use of cluster munitions. Countries that are not yet signatories of the Convention on Cluster Munitions should sign this international treaty. To mitigate the risk of cluster munitions, civilians should not be allowed to return to lands contaminated, or suspected to be contaminated, with cluster munitions, before specialized agencies clear the lands from munitions and other unexploded remnants of war. Military forces who use these weapons should indicate the areas where cluster munitions are used, to assist in demining lands and decrease civilian casualties. The international community should fund and support demining projects, with support from producers and users of cluster munitions. Since injuries due to cluster munitions often lead to functional impairment, victims of submunition blasts should be given access to mental health services. Counselling and rehabilitation programmes would also support the social reintegration of affected individuals. Awareness raising on cluster munitions should be provided to affected populations. Children in particular need to be made aware of the risks of cluster munitions, because many submunitions have intriguing shapes and colours, increasing the risk of children picking them up and being injured.

In addition, further research is needed on the type of physical injuries, psychological impact, socioeconomic effect and environmental repercussion of cluster munition use. Findings would further support the ban on cluster munitions and encourage more countries to join the convention.
